# Safety and effectiveness of bycross rotational atherectomy and aspiration device: a prospective, multi-center pre-market approval study

**DOI:** 10.1186/s42155-023-00363-0

**Published:** 2023-03-29

**Authors:** Jörg Tessarek, Ralf Kolvenbach

**Affiliations:** 1 Department of Vascular Surgery in Bonifatius Hospital, Lingen, Germany; 2 Department of Vascular Surgery in Sana Kliniken, Düsseldorf Gerresheim, Germany

**Keywords:** Rotational atherectomy, Femoropopliteal calcification, Stenosis, Restenosis, Peripheral arterial disease, Superficial femoral, Popliteal revascularization, Rotational, Aspiration thrombectomy, Embolic protection BYCROSS®

## Abstract

**Purpose:**

To demonstrate safety and effectiveness of the novel ByCross® atherectomy system for treatment of complex femorodistal > 80% arterial stenosis.

**Materials and Methods:**

From September 2018 until April 2019 39 patients with 41 femorodistal lesions including the femoropopliteal and distal popliteal segments were treated in a prospective, nonrandomized pre-market approval study with 6 months follow up using the ByCross® atherectomy device (ClinicalTrials.gov identifier NCT03724279). Adjunctive treatment with balloon dilatation or stenting was allowed by the protocol. Mean patient age was 72 years with 62% male. The average lesions length was 125 ± 118 mm (30 and 450 mm) with an average reference vessel diameter of 5.2 ± 0.85 mm and a mean stenosis of 96.4 ± 6.2% based on CT or MR angiography measurements. The primary performance endpoint was defined as acute procedural success with angiographically determined residual stenosis of ≤ 50% and of ≤ 30% after atherectomy or adjunctive treatment. The primary safety endpoint was the major adverse event (MAE) rate through 30 days. Secondary endpoints were stenosis of the target lesions measured by duplex ultrasound (DUS) and the ankle-brachial pressure index (ABPI) at discharge, 30 and 180 days as well as any major adverse event (MAE) through 6 months.

**Results:**

The acute procedural success was achieved in 39/41 (95.12%) lesions, partially without wire guidance (11/41 (26.82%)). No embolic protection was used, and adjunctive angioplasty and stenting was performed in 40/41 (97.56%) and 12/41 (29.26%) lesions, respectively without device related MAE’s at 30 days. Mean level of stenosis was 5.7% at discharge and 21.7% at 6 months FU measured by DUS. Mean ABPI was 0.8, 1.0 and 0.8 at baseline, discharge, and 6 months FU respectively.

**Conclusions:**

Based on the high technical success rate and the low rates of MAE`s through six months, the BYCROSS® Atherectomy System has shown to be safe and effective for the crossing and atherectomy of complex lower-extremity arterial occlusions.

**Trial registration:**

October 17, 2018,retrospectively registered. ClinicalTrials.gov Identifier: NCT03724279; https://clinicaltrials.gov/ct2/show/record/NCT03724279

## Introduction

Peripheral artery disease (PAD) is a common manifestation of systemic atherosclerosis with variable clinical scenarios increasing in many societies and meanwhile PAD represents a considerable challenge for the social and health care systems (Bisdas et al. 2016; Chaar et al. 2017).

Treatment options are conservative treatment with training and best medical treatment. Beside surgical bypass, for the vast majority of occlusive lesions endovascular intervention has become the gold standard instead of bypass surgery (Criqui and Aboyans 2015; Davis et al. 2017).

This includes percutaneous transluminal balloon angioplasty (PTA) as a standalone solution or PTA with additional stenting. Furthermore, atherectomy in combination with adjunctive PTA with drug eluting balloons has been propagated for drug mediated suppression of the physiologic response after evasion of the occlusive material (Franzone et al. 2012).

Meanwhile, various atherectomy systems are available including directional plaques excision, laser atheroablation, rotational aspiration/atherectomy and orbital atherectomy (Freitas et al. 2017).

Severely calcified lesions still represent the very challenge for endovascular treatment (Gehani and Mees 1998). Debulking of plaques material with aspiration of debris and thrombus might help to overcome the limitations of simple PTA, thus potentially reducing the need for adjunctive stenting and the rate of re-stenosis (Katsanos et al. 2017).

Llow rotational speed (2000-4500 rpm), the variable tip size up to 4.7 mm and pump mediated aspiration, as three of the main characteristics of the novel BYCROSS® system (Taryag Medical Ltd., Or Akiva, Israel), were thought to increase the overall safety and effectiveness in terms of embolic complications and initial lumen gain.

The currently available atherectomy systems provide distinct approaches but with device specific limitations. The major concerns are the need for wire passage prior to atherectomy with the risk of subintimal wire position and, having no adjunctive aspiration capacity, the risk of embolization. The robustness of cutting devices rotating at high speed result in significant mechanical stress at the vessel wall and the risk of dissection, vessel perforation, distal embolization and immediate initiation of physiologic repair mechanisms (Maehara et al. 2015; McKinsey et al. 2014; Ostchega et al. 2007; Rocha-Singh et al. 2021; Rocha-Singh et al. 2014)

The reported embolization rates in the range of 2% to 4% (Sauguet et al. 2019) resulted in the strong recommendation to use embolic protection devices for some of the devices.

We present the data from the German CE trial of the BYCROSS® Atherectomy System. The study endpoints for the CE approval study were identical with those from other atherectomy studies to allow comparison of the technical and clinical results after 6 months follow up. The combination of low velocity atherectomy with variable tip diameter and high aspiration capacity in a single device makes embolic protection or device exchange for more lumen gain redundant. The ByCross® is the first atherectomy that can achieve lesion passage without initial wire crossing, which represents a technically novel approach to atherectomy.

## Materials and methods

### Trial design

The prospective, two-center, non-randomized, single- arm trial (*ClinicalTrials.gov* NCT03724279) was conducted to evaluate the safety and effectiveness of the novel BYCROSS® system in the percutaneous treatment of de novo atherosclerotic lesions in the femoropopliteal segment and below the knee segments with a minimum diameter of 3 mm excluding the last 10 mm of the tibioperoneal tract. Performance and safety goals were pre-determined for evaluating sample size and defining statistical goals based on previous reports of other atherectomy trials. The study was approved by the local ethic committees and closely surveilled by the German Federal Institute of Drugs and Medical Devices (BfarM), with eight interim reports about progress, events, results, and completion after each of the first eight cases followed by 48 h event free follow up before recruitment could proceed.

### Statistical analysis

The expected Acute Procedural Success rate was 95% based on literature reporting of other atherectomy techniques (Ostchega et al. 2007; Scheinert et al. 2018; Shammas 2017).

The lower confidence limit greater than or equal to 85% and taking into account a dropout rate of 5% required a sample size of 42 lesions to be enrolled. Demographic and baseline condition related characteristics are tabulated and summarized. Continuous variables are summarized by a mean, standard deviation, minimum, median and maximum, and categorical variables by a count and percentage. A count and percentage of subjects with Acute Procedural Success are calculated and presented with a one-sided 95% exact binomial confidence interval.

### Device description

The novel BYCROSS® Atherectomy system is a 6F disposable, rotational atherectomy and thrombectomy device with two rotational velocities (2000 rpm-4500 rpm). The battery can be depolluted separately. The rotating shaft, available with 700 mm and 950 mm length at the time of the study, uses the Archimedes screw principle for debris transport in addition to a pump in the handle. The pump shows an aspiration capacity of 65 cc per minute in combination with a 6F sheath and 200 cc in combination with an 8F sheath.

BYCROSS® has a front- and side cutting metal tip at the distal end of the shaft with an elastic Nitinol wing that can asymmetrically bow and enlarge the tip diameter from 1.9 mm to 4.7 mm. The system is steered by a microchip adapting the battery force to the needs for advancing the tip. Reversed rotation is also possible.

The BYCROSS® does not require wire passage through the lesion prior to atherectomy. The tip design is dedicated for crossing and debulking of stenotic lesions and total occlusions independent from the calcium burden while the aspiration force aims at effective debris aspiration independent from its morphology. As soon as contact between tip and target lesion is achieved the system moves actively forward. The wire placement for adjunctive therapies can be performed from the rear after passage of the lesion. Contrast injection is possible via the sheath or the shaft.

The low rotational speed prevents heating and carbonization inside the shaft and reduces the risk of thermal damage to the vessel wall. The maximum tip temperature after 30 s of drilling in dry bone equivalent in lab testings was 47.1 °C and the environmental temperature rise did not exceed 40.1 °C (data available by TARYAG Inc., Israel), Therefore the device has no run time limitation.

The BYCROSS® system was available with either 95 cm and 70 cm shaft length for the study requiring a 90 cm or 65 cm sheath for guidance.

The trial was performed in accordance with the Declaration of Helsinki, the EC Directive (93/42/EEC art.15) and local Member States transpositions. It also followed the guidelines for conducting a Clinical Investigation as outlined in the European Harmonized Standard, EN ISO 14155:2011(E), and in accordance with the principles of ICH GCP. Two centers in Germany participated after receiving approval from their independent, local medical ethics committees. Preparation for the study was identical for both centers.

All participants had noninvasive diagnostic imaging with CTA or MRA prior to intervention and were recruited after given informed consent. Exclusion criteria were recurrent or in stent lesions, a vessel diameter below 3 mm and contraindication for platelet inhibiting therapy or a life expectancy below 12 months. Inflow lesions in the iliac arteries could be treated according to the protocol. The partially extreme calcium burden of the 30 to 450 mm long lesions was measured and documented according to the peripheral artery calcification severity score (PACSS score) (Shammas et al. 2011). A high PACSS score of 4 or 5 describing long, circumferential and lumen prominent calcium deposits, did not represent an exclusion criterion as in other trials. Eligibility criteria are summarized in Table 1.

**Table 1:** Eligibility criteria for enrollment

Inclusion criteria
Age ≥ 18 Subject with documented symptomatic PAD (Rutherford 2–6) with de novo lesion eligible for percutaneous intervention Target lesion is at least 10 mm distal to the SFA origin and at least 10 mm proximal to the distal end of the TPT Degree of stenosis ≥ 80% based on CTA, MRA or angiography Vessel lumen diameter ≥ 3.0 mm Lesion length ≥ 3.0 cm Subject has been informed on the nature of the study and is willing and able to provide informed consent Subject is capable of meeting study requirements including presences at follow-up visits
Exclusion criteria
Subject is unable to take antiplatelet drugs or anticoagulation Vessel of the cardiopulmonary, coronary or cerebral circulation Subject has anticipated life expectance < 12 month Subject is diagnosed with impaired renal function (creatinine > 2.5 mg/dL) Subject has undergone or planned surgical or endovascular procedure 15 days before or after the study procedure Vessel lumen < 3.0 mm Stent at access and target vessel or In-stent restenosis at target lesion Target and/or access vessel includes by-pass graft Target vessel is dissected Target is at vessel segment which includes tortuous course with radius of curvature < = 40 mm Access pathway includes tortuous course with radius of curvature < = 25 mm Target and/or access vessel includes aneurysmatically altered segments Persistent vasospasm Known or suspected allergy to any of the components of the system or to a medicinal product to be administered in connection with the planned procedure Subject is pregnant or planning to become pregnant within the study period, or lactating mothers Subject is enrolled to another clinical investigation that might interfere with this study

From September 2018 until April 2019, 39 out of 44 screened patients were enrolled with 42 lesions out of which 41 lesions treated according to the protocol, 25 patients (59.5%) at Augusta and 16 (40.5%) at Bonifatius. The patient characteristics are listed in Table 2. Since full-analysis compared with the per-protocol-analysis included one single lesion, which was not treated with the investigation device due to technical failure, from this point forward this report discussed the per-protocol-analysis only. Patients’ demographics and risk factors are presented in Table 2. According to the conditions determined by the BfArM as first-in-man study (the first 8 subjects were successively treated with the demand of an uneventful interim phase of 48 h before the next procedure (Initial study phase). Progress of the study was allowed after the primary performance endpoint, defined as uneventful 30 day follow up, had been met for the eight initial phase patients. Then, 34 further study procedures were performed independent from any timeline.

**Table 2:** Demographics, history and risk factors (*N* = 39 patients/42 lesions) *

Age (years)	72 ± 9.82 (43–86)
Sex – Male, %	26 (62)
Body Mass Index (Kg/m2)	27.1 ± 3.8 (20.2–38.5)
Diabetes, %	13 (33)
High Cholesterol, %	12 (31)
Hypertension, %	34 (87)
Smoking, %	21 (54)
Currently smoking, %	17 (44)

^*^(continuous data are presented as the means ± standard deviation (range), categorical data are given as the counts (percentage))

The procedures were performed percutaneously with either antegrade or contralateral access, depending on the lesion morphology.. Any lesion ≥ 80% stenosis with a minimum distance of 10 mm below the femoral bifurcation and 10 mm above the bottom end of the tibioperoneal tract could be recruited.

Per protocol the target lesion and the runoff were evaluated angiographically prior to BYCROSS® insertion, after atherectomy and after adjunctive treatment if it was regarded as necessary to achieve the 30% residual stenosis outcome.

### Study endpoints and follow-up

The primary performance endpoint was Acute Procedural Success defined as ≤ 50% residual stenosis after atherectomy alone and residual stenosis ≤ 30% in the completion angiography achieved by either atherectomy, angioplasty and/or stenting. “Successful” also included the absence of any Serious Adverse Events (SAE) during the procedure. Primary safety endpoint was lack of Major Adverse Events (MAE)*, at 30 days follow-up. MAE’s were defined as:

- amputation (transmetatarsal or higher)
- distal embolization requiring treatment
- ex-procedure TLR (Target Lesion Revascularization) and TVR (Target Vessel Revascularization)
- myocardial infraction
- severe bleeding
- arterial perforation
- pseudoaneurysm/Arteriovenous fistula
- acute renal failure
- acute arterial closure and any other Adverse Event that required surgery
- unanticipated additional vascular repair and/or transfusion and/or intravenous antibiotics
- extended hospitalization (> 24 h over expected) and readmission to hospital between 24 h after discharge and 30 days post procedure.

Secondary performance endpoints included deterioration of ABI and lumen loss (restenosis) at lesion site after 30 and 180 days follow up determined with DUS. Secondary safety endpoint was any documented Major Adverse Events (MAE)* during the index procedure, at hospital discharge and at 6 months follow-up.

Lesion assessment at baseline is summarized in Table 3. All patients had noninvasive diagnostic imaging prior to treatment. Patients and lesions were re-assessed at discharge, 30 days and 6-month post procedure. At each follow up visit medications were recorded in the eCRF medication log. Blood samples taken at each FU visit included blood count and coagulation status. ABI and degree of target lesion stenosis were also measured by doppler and Duplex ultrasound.

**Table 3:** mean lesion characteristics in the two participating centers

	Augusta	Bonifatius	All
Target Vessel			
SFA	20	8	28
SFA-POP	3	8	11
POP	2	–	2
Lesion length (mm)	54	229	125
Vessel Diameter (mm)	5.2	5.2	5.2
PACSS Grade	3.5	2.5	3.1

## Results

Two lesions were screened but not enrolled since their angiographies displayed lesion stenosis of < 80%. Only one patient was lost to FU and evaluation of safety primary endpoint.

The intraprocedural lesion characteristics and data are summarized in Table 4 with lower one-sided 95% exact confidence limits. Mean PACSS score was 3.1 ± 1.4, lumen diameter ranged between 4 and 8 mm with mean of 5.2 ± 0.85, and lesion length between 30 and 450 mm with mean of 124.7 ± 118.

**Table 4:** Intra procedure lesion and procedure data

PACSS grade	3.1 ± 1.4 (0.0–4.0)
Vessel diameter (mm)	5.2 ± 0.85 (4.0–8.0)
Lesion length (mm)	124.7 ± 118.43 (30.0–450.0)
Post-atherectomy PTA	95.12% 39/41
Post atherectomy stenting	29.26% 12/41
Pre-procedure stenosis (%)	96.4% (80–100)
Post ByCross stenosis < 50%	95.12% 39/41
ByCross passage without guidewire	26.8% (11/41)
Post-procedure stenosis (%)	5.85 ± 16.2 (0–100)

No pre-treatment with PTA of lesions was required. Post-atherectomy PTA as adjunctive therapy was used in 95.12% (39/41). Stenting was used after PTA in 28.5% (12/41). All the patients were free from device related MAE at 30-days post procedure. 95.12% (39/41) of the patients met the criterion for acute procedural success, the lower limit of the one-sided 95% exact CI was 86.29%.

### Primary endpoint sub-group analysis

No gender related nor target segment differences could be observed in the subgroup analysis. The outcomes appear better for lower pre-treatment stenosis levels (80–99) and for lower baseline PACS scores (0–3), but due to the small cohorts, there is no statistical significance (Table 5). The DUS based measurement of the vessel lumen at discharge, 30 and 180 days showed a slight deterioration of vessel lumen with a mean residual stenosis of 5.7% at discharge, 8.9% at 30 days and 21.7% (0.0–34.6%) at 180 days. Comparing the lesions in terms of lesion length, a slight difference in favor of the short ones (mean length 53.6 mm vs. 229.2 mm) with a 180 day mean stenosis of 16.7% vs. 21.7% was detectable, as expected.

**Table 5:** Primary endpoint subgroup data

Data	Performance met
Gender	
Male	92.3% (24/26)
Female	93.75% (15/16)
Target Vessel	
SFA	93.1% (27/29)
SFA-POP	90.9% (10/11)
POP	100% (2/2)
Stenosis – Pre	
100%	90% (27/30)
85–99%	100% (12/12)
PACSS Grade	
4	88.9% (24/27)
0–3	100% (15/15)
Rutherford class^a^	
Class 2	5.88% (1)
Class 3	17.64% (3)
Class 4	47.05% (8)
Class 5	29.41% (5)

^a^ Rutherford classification available only from the Bonifatius hospital cohort

### Secondary endpoint safety analysis

Table 6 presents the secondary safety endpoint at 6-month FU. There were no device-related adverse events. Two patients developed acute occlusions of the target lesion > 48 h after the procedure treated by surgical thrombectomy with uneventful follow up. One patient showed severe stenosis at the ipsilateral common femoral artery in the pre-discharge DUS examination. The lesion at the access vessel was treated with directional atherectomy from the contralateral side. One patient developed acute occlusion of a distal bypass (popliteo-politeal) with patent lesion of the proximal femoral artery. One patient presented with progredient claudication with a severe stenosis of the contralateral access vessel resulting in surgical atherectomy.

**Table 6:** Secondary safety endpoint data

Adverse events count:	
Post-procedure SAE/MAE	0% (0/41)
Discharge day SAE/MAE	9.5% (4/41)
30-Day SAE/MAE	0% (0/41)^a^
6-Month	2.4% (1/41)^a^
Adverse events type	
Restenosis of target lesion	2
Restenosis of target vessel	2
Restenosis of another vessel	1

^a^ 30-Day and 6-Month follow up does not include to subject who was lost to follow up

### Secondary endpoint performance analysis

Table 7 present descriptive statistics of the secondary efficacy endpoints ABPI and stenosis % as well as the change from baseline at each visit. Model estimated mean (LSmeans) changes from baseline for ABPI and stenosis % respectively with level of significance and 95% confidence intervals (CI), post procedure, after 30-days and after 6-months is as well presented. A reduction was observed in both ABPI and vessel lumen at each of the visits. Change in ABPI was found significant from discharge to base line (*P* < 0.001) and from 30-Day follow up to baseline (*P* = 0.0396), but not from 6-Month follow up to baseline (*P* = 0.874). With respect to stenosis change from discharge and follow up to baseline was found to be significant for all time points (*P* < 0.001).

**Table 7:** Secondary performance endpoint data

ABPI:	Actual	Change from baseline	*P*-value
Baseline	0.8 ± 0.36 (0.3–2.0)	–	
Discharge	1 ± 0.32 (0.9–2.0)	0.2 ± 0.8 (-0.3–0.7)	< 0.001
30-Day	0.9 ± 0.25 (0.3–1.4)	0.3 ± 0.26 (-0.2–0.7)	0.0396
6-Month	0.8 ± 0.27 (0.3–1.5)	0.0 ± 0.4 (-1.5–0.7)	0.8704
Stenosis (%)			
Baseline	96.4 ± 6.18 (80–100)		
Discharge	5.7 ± 17.26 (0–100)	-90.5 ± 17.6 (-100–0)	< 0.001
30-Day	8.9 ± 27 (0–100)	-87.6 ± 26.6 (-100–0)	< 0.001
6-Month	21.7 ± 34 (0–100)	-74.8 ± 33.1 (-100–0)	< 0.001

The Rutherford classification as documented outside the protocol (Bonifatius Hospital Lingen) changed significantly within the 6-month FU period. At 6 months only two patients out of 16 were in class 2 and 3 coming from 5 or 4 at baseline.

## Discussion

**Fig. 1:**
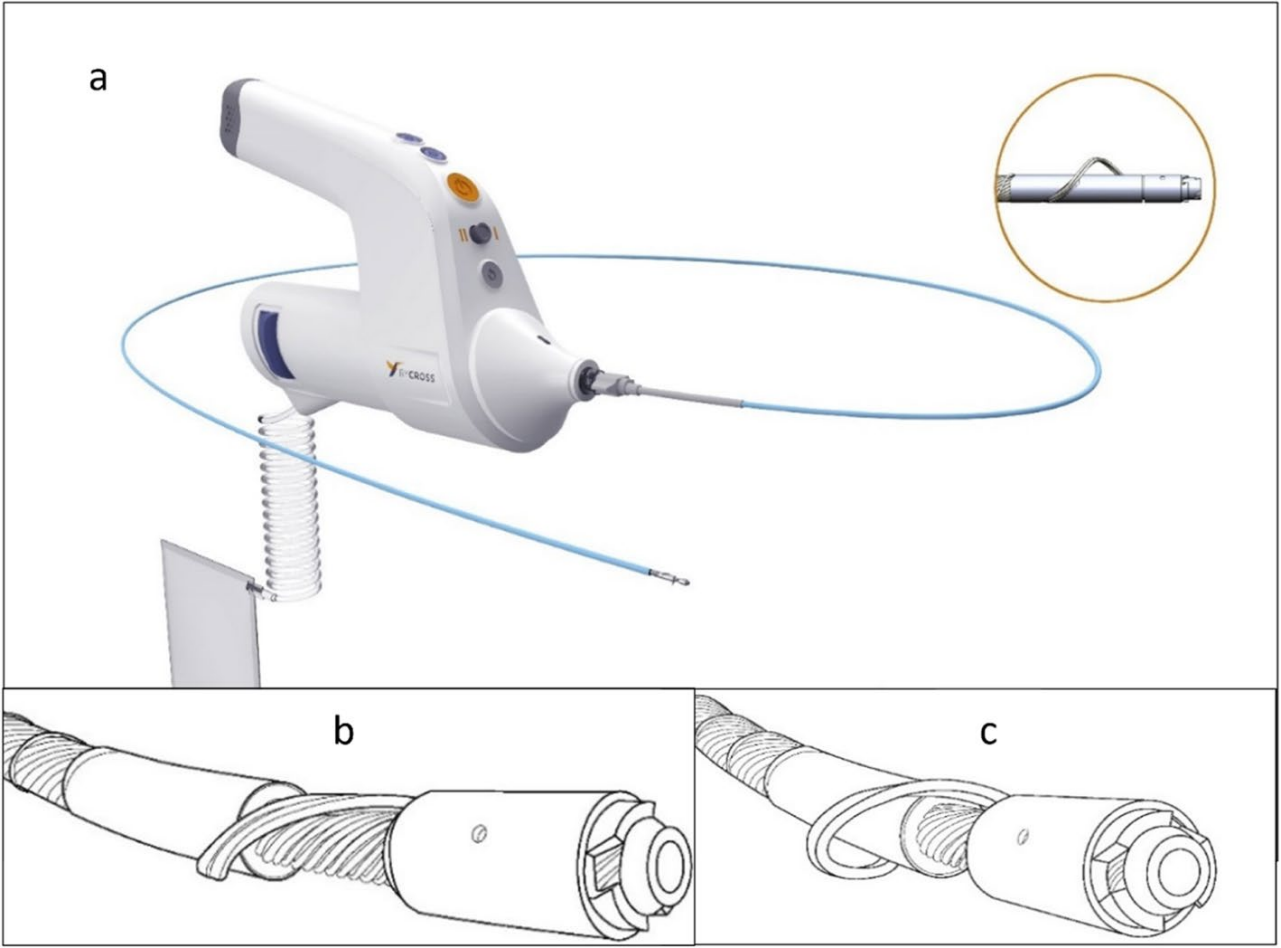
**a** ByCross device with attached 6F sheath, (**b**) ByCross tip closed, (**c**) ByCross tip expanded

Endovascular atherectomy by either directional or rotational systems has developed as an alternative method since 1990 (McKinsey et al. 2014; Ostchega et al. 2007; Shammas et al. 2011; Shammas et al. 2016) especially for lesions being resistant to simple PTA or stenting (Katsanos et al. 2017; Shrikhande et al. 2011) or for mobile vessel segments where stenting might cause early restenosis (Fig. 1)

The evasion of plaques material and partially also the inner layers of the vessel wall allows effective lumen gain and the reduction of biologically active components, that are repsonsible for the immediate physiological response with restenosis initiation. The wider post atherectomy lumen also allows to postdilate with lower inflation pressure to achieve good results thus reducing further barotrauma to the vessel wall. On the other hand, all atherectomy modalities increase the risk of distal embolization (Stavroulakis et al. 2017) with an eventrate up to 22% in some surveys vs. 0.9% for PTA alone. (Stavroulakis et al. 2018)

Furthermore, the robustness of the devices with sharp cutting edges rotating at high speed (40-190 K rpm) for plaques demolition increases the risk of mechanical and thermal vessel injuries^(25)^, especially when the guidewire should go subintimal in long occlusions.

**Fig. 2:**
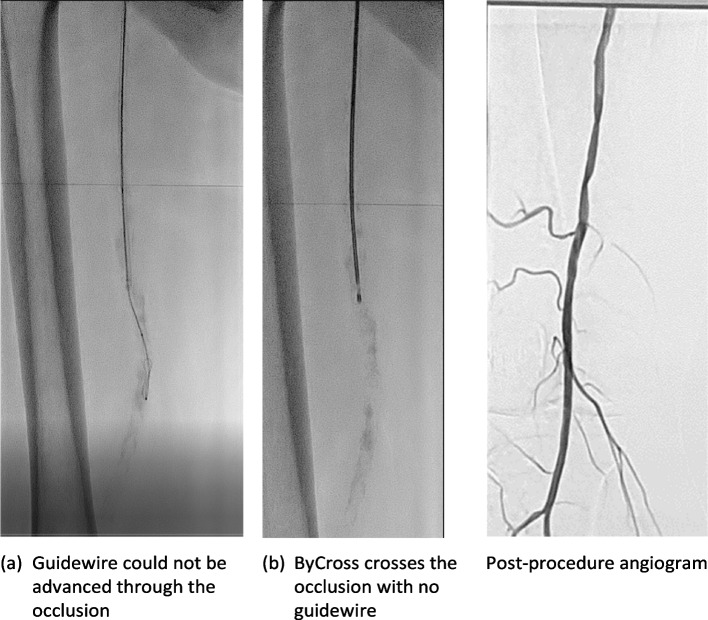
Recanalization of a severely calcified femoral artery occlusion without wire guidance. Adjunctive treatment with ballooning was performed

The novel rotational atherectomy system BYCROSS® represents a novel approach in terms of rotational speed and the combination of atherectomy and thrombectomy device with high aspiration capacity and no need for embolic protection. The low rotational speed and the dual transport system showed to be effective in preventing embolic events, which did not occur. The design of the tip similar to a masonry drill allowed to pass 11 lesions without initial wire guidance (Fig. 2).

The results from the trial have shown that the device is highly effective with a 95.12% technical success rate with lower confidence limit greater than or equal to 85% and no intraprocedural MAE`s or SAE`s. In particular, there were no device related SAE/MAE`s. The success rate from BYCROSS® is similar to those of the EASE trial with the Phoenix® device (Philips b.v., Best, The Netherlands), also showing a success rate of 95.1% but a MAE rate 16.8% through six months vs. 2.4% in this study, noticing, that the treated lesions in the two studies are not comparable. The EASE cohort had a mean lesion length of 34 mm vs. 124.9 mm and a mean stenosis of 89.5% vs. 96.4% in the BYCROSS® study (Shammas 2017).

The primary performance endpoint, here the passage of the occlusion by the BYCROSS® device with a post atherectomy residual stenosis ≤ 50% (assessed by angiography) and complete procedural success with a residual a residual stenosis ≤ 30% after adjunctive therapy with angioplasty and/or stenting if required, with no Serious Adverse Events (SAE) during the procedure was met at 39/41 lesions. Primary patency (stenosis ≤ 50%) at 6 months FU was observed at 34/39 lesions, i.e. 87.2% while the six-month TLR and TVR were 88.0% and 86.1% for the Phoenix™ rotational atherectomy device (Royal Philips b.v., The Netherlands) for much shorter lesions. The intraprocedural embolization rate in the BYCROSS® trial was 0% independent from lesion calcification and length without any distal protection system used. In the EASE trial ^(16)^ the rate of distal embolization was 1% and in the DEFIFINITE-LE trial with the SilverHawk and TurboHawk devices (Medtronic Inc., USA) 3.8% with distal protection ^(19)^.

The performance goal with primary and secondary safety endpoints counting all postprocedural Major Adverse Events (MAE) and SAEs at 30 and 180 days follow-up, was also met independent from gender, length of the lesion and degree of stenosis as well as the PACS score at a very low rate of MAE/SAE at 30 days (0%) and 6 month FU (2.4%).

Although the lesion complexity with up to 450 mm length and a mean degree of stenosis of 96.4% in a patient set with more than 77% presenting with CLI (Rutherford IV and V, subset of Bonifatius Hospital**)** was extreme no embolization was seen in the obligatory angiographic imaging of run off vessels or clinically evident.

The overall stenting rate of 29.26% can be regarded as low in this setting compared to other studies showing a provisional stenting rate of 53% (Pölnitz et al. 1990). The shorter lesions (mean length 53.6 mm) had the higher PACS score. 92% (*n* = 23) were postdilated but only 12% (*n* = 3) required stenting. The longer lesions (mean length 229 mm) required PTA in 94.1% (*n* = 16) and focal stent application in 52.96% (*n* = 9). But in the vast majority of long lesions only spot stenting was used for flow relevant dissections or focal residual stenosis. Complex lesion morphologies with severe calcification as included in this trial have remained unstudied as they were excluded from randomized studies (Zeller et al. 2007).

In the study low pressure ballooning after atherectomy with 2–4 atmospheres was sufficient for a complete balloon expansion to nominal diameter and for meeting the performance goal or less than 30% residual stenosis. No drug coated or eluting devices were used although several publications have promoted this in combination with atherectomy to reduce the focal physiologic repair mechanisms (Zeller et al. 2007; Zeller et al. 2009).

The clinical outcome after 6 months, although not an endpoint in the protocol, also showed significant improvements in the Rutherford documentation of the Bonifatius Hospital subgroup.

The novel BYCROSS® system also has several advantages when compared to other devices on the market. According to the IFU, it is not limited to the femoropopliteal segment or de novo stenosis and can also be used in aorto-iliac recanalization or in-stent lesions with or against the blood flow. It does not need blood cooling due to the low rotational velocity thus making any infusion mechanism redundant. It is a disposable without costs for investment and it can be connected to variable sheath diameters from 6F-8F thereby increasing the aspiration capacity from 65 to 200 cc per minute and making additional embolic protection devices redundant with considerable cost savings.

## Conclusion

BYCROSS® could show the effectiveness and safety for revascularization of complex femoropopliteal lesions. The low rotational velocity and the tip design allow for wireless passage of lesions making complex procedures for wire placement in the distal lumen first redundant. The combination of three functions in a single device (crossing devie, atherectomy with variable tip diameter and aspiration thrombectomy) represents a novel and promising atherectomy device with outstanding features, i.e. the option for wireless passage of complex lesions with a variable tip diameter and a low stenting rate. The immediate and midterm results for complex lesions up to 450 mm are promising but require further evaluation in a wider setting type with operators of different experience level. Further data from the real-world setting and long term follow up are mandatory to completely realize the BYCROSS® related advances in atherectomy technique driven revascularization.
