# Correction: Safety and effectiveness of bycross rotational atherectomy and aspiration device: a prospective, multi-center pre-market approval study

**DOI:** 10.1186/s42155-023-00375-w

**Published:** 2023-05-10

**Authors:** Jörg Tessarek, Ralf Kolvenbach

**Affiliations:** 1 Department of Vascular Surgery in Bonifatius Hospital, Lingen, Germany; 2 Department of Vascular Surgery in Sana Kliniken, Düsseldorf Gerresheim, Germany

**Correction: CVIR Endovasc 6, 19 (2023).**

10.1186/s42155-023-00363-0.

Following publication of the original article [1], the editor has identified an error in the reference citations. The citations were xxx instead of the actual year in the following references: McKinsey et al. and Sauguet et al.

The original article has been updated.
